# LaSSO, a strategy for genome-wide mapping of intronic lariats and branch points using RNA-seq

**DOI:** 10.1101/gr.166819.113

**Published:** 2014-07

**Authors:** Danny A. Bitton, Charalampos Rallis, Daniel C. Jeffares, Graeme C. Smith, Yuan Y.C. Chen, Sandra Codlin, Samuel Marguerat, Jürg Bähler

**Affiliations:** University College London, Department of Genetics, Evolution and Environment, London WC1E 6BT, United Kingdom; University College London, UCL Cancer Institute, London WC1E 6BT, United Kingdom

## Abstract

Both canonical and alternative splicing of RNAs are governed by intronic sequence elements and produce transient lariat structures fastened by branch points within introns. To map precisely the location of branch points on a genomic scale, we developed LaSSO (Lariat Sequence Site Origin), a data-driven algorithm which utilizes RNA-seq data. Using fission yeast cells lacking the debranching enzyme Dbr1, LaSSO not only accurately identified canonical splicing events, but also pinpointed novel, but rare, exon-skipping events, which may reflect aberrantly spliced transcripts. Compromised intron turnover perturbed gene regulation at multiple levels, including splicing and protein translation. Notably, Dbr1 function was also critical for the expression of mitochondrial genes and for the processing of self-spliced mitochondrial introns. LaSSO showed better sensitivity and accuracy than algorithms used for computational branch-point prediction or for empirical branch-point determination. Even when applied to a human data set acquired in the presence of debranching activity, LaSSO identified both canonical and exon-skipping branch points. LaSSO thus provides an effective approach for defining high-resolution maps of branch-site sequences and intronic elements on a genomic scale. LaSSO should be useful to validate introns and uncover branch-point sequences in any eukaryote, and it could be integrated into RNA-seq pipelines.

Introns and exons refer to noncoding and coding sequences, respectively, that constitute protein-coding genes (Gilbert 1978). To create a functional messenger RNA (mRNA), introns are excised via a highly conserved and accurate process called splicing that culminates in concatenation of exon sequences into translatable transcripts. Splicing entails two transesterification reactions catalyzed by the spliceosome, a large RNA-protein complex (Wahl et al. 2009). The first reaction involves a nucleophilic attack of an adenosine (branch point) on the 5′-splice donor, resulting in a lariat structure fixed by a 2′–5′ phosphodiester bond; the intron remains only attached to the downstream exon (Fig. 1A,B; Padgett et al. 1985). The second reaction involves an attack of the detached upstream exon on the 3′-splice acceptor, resulting in intron lariat release and exon ligation (Fig. 1C). The intron is then processed by exonucleolytic cleavage of the 3′-lariat tail and linearization by the debranching enzyme Dbr1 (Fig. 1D; Kim et al. 2000; Cheng and Menees 2011). The spliceosome is disassembled and recycled (Arenas and Abelson 1997; Martin et al. 2002). Lariat debranching is a rate-limiting step for intron degradation or further processing (Fig. 1E), and lariats accumulate in cells with compromised debranching activity (Fig. 1F; Nam et al. 1997; Kim et al. 2000; Ye et al. 2005).

**Figure 1.**
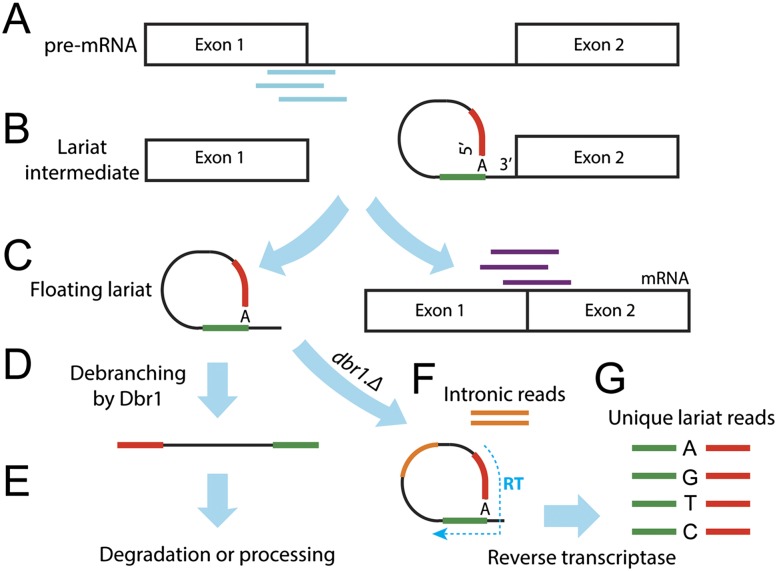
Scheme of intron splicing and diagnostic sequence reads. (*A*) Pre-mRNA with diagnostic exon-intron reads (cyan). (*B*) First transesterification reaction: lariat intermediate with phosphodiester bond between 5′ splice donor (red) and branch-point adenine (A) along with upstream sequence (green). (*C*) Final splicing reaction: exons are ligated yielding mature mRNA with diagnostic exon-exon junction reads (purple), while the lariat is excised. (*D*) Intron 3′ tail removal and debranching. (*E*) Rapid degradation or further processing. (*F*) In *dbr1Δ* cells, lariats become stabilized and accumulate, resulting in enhanced intronic sequence reads (orange). The reverse transcriptase also reads through the 2′–5′ linkage (hatched blue arrow). (*G*) This reverse transcription produces unique lariat reads, where the sequence upstream of the branch point (green) precedes the 5′ segment of the intron (red). The enzyme often mutates the branch-point adenine to any other nucleotide as illustrated. The accumulation of lariat structures would inevitably result in the production of additional intronic reads (orange) that enhance intronic expression level.

There is a growing appreciation that introns exert a broad spectrum of cellular roles (Chorev and Carmel 2012). Functional RNAs can be transcribed within introns, including small nucleolar RNAs (snoRNAs) (Ooi et al. 1998), which depend on correct intron processing. Splicing plays critical roles in gene regulation (Wang et al. 2008; Cheng and Menees 2011) and augments proteome diversity via alternative splicing (exon skipping or intron retention) (Wang et al. 2008). Aberrant splicing is implicated in a wide range of human diseases (Cooper et al. 2009). Despite their importance, many introns await characterization; thus there is a need for methods to better define splicing events and intronic branch points on a genome-wide scale.

Introns contain sequence elements required for correct splicing, including splice donor and acceptor sites, the branch site (usually adenine as the branch-point base) (Padgett et al. 1985), polypyrimidine tracts, and intronic splicing enhancers or silencers (Yeo et al. 2007; Wang and Burge 2008). The branch-site is essential for spliceosome assembly and accurate removal of the intron. To date, only tens of branch-sites have been experimentally characterized (Kol et al. 2005; Gao et al. 2008), reflecting a lack of high-throughput methods to analyze them. Lariat detection is often based on RT-PCR that exploits the ability of the reverse transcriptase to read through the branch-site (Ohi et al. 2007). A recent study used RNA-seq data to identify unique lariat reads (Taggart et al. 2012), albeit only slightly more than 2000 such reads were identified among 1.6 billion total reads, allowing validation of 759 human introns. Another study has sequenced 2D-gel-purified lariats of fission yeast, leading to enhanced intron detection based on a laborious lariat-isolation protocol (Awan et al. 2013). Computational branch-site predictions, on the other hand, are based on pattern-search algorithms (Drabenstot et al. 2003) and can consider comparative genomics data (Kol et al. 2005). Predictions rely on assumptions about branch-point positions, so require a priori knowledge while lacking large, experimentally validated data.

Here we present a data-driven method that precisely locates branch points on a global scale. The algorithm LaSSO (Lariat Sequence Site Origin) builds a database of all possible lariat signatures from all known introns, including those that could be generated from all possible exon-skipping events. LaSSO considers every base in a given intron as a potential branch point. RNA-seq data are then searched against this lariat database to locate branch points that are supported by sequence reads. We validate our approach using fission yeast (*Schizosaccharomyces pombe*), which provides a powerful system for splicing studies. About 47% of its genes are spliced via conserved splicing signals and factors, including SR-like proteins implicated in alternative splicing and the debranching enzyme Dbr1 (Käufer and Potashkin 2000; Kim et al. 2000). Fission yeast cells lacking Dbr1 (*dbr1Δ* deletion mutant) are slow growing but viable (Nam et al. 1997), providing a straightforward genetic approach to enrich for lariats. We also show that LaSSO is effective in defining branch points in human introns, even in the presence of debranching activity.

## Results

### Transcript and intronic expression signatures in *dbr1Δ* cells

**Figure 2.**
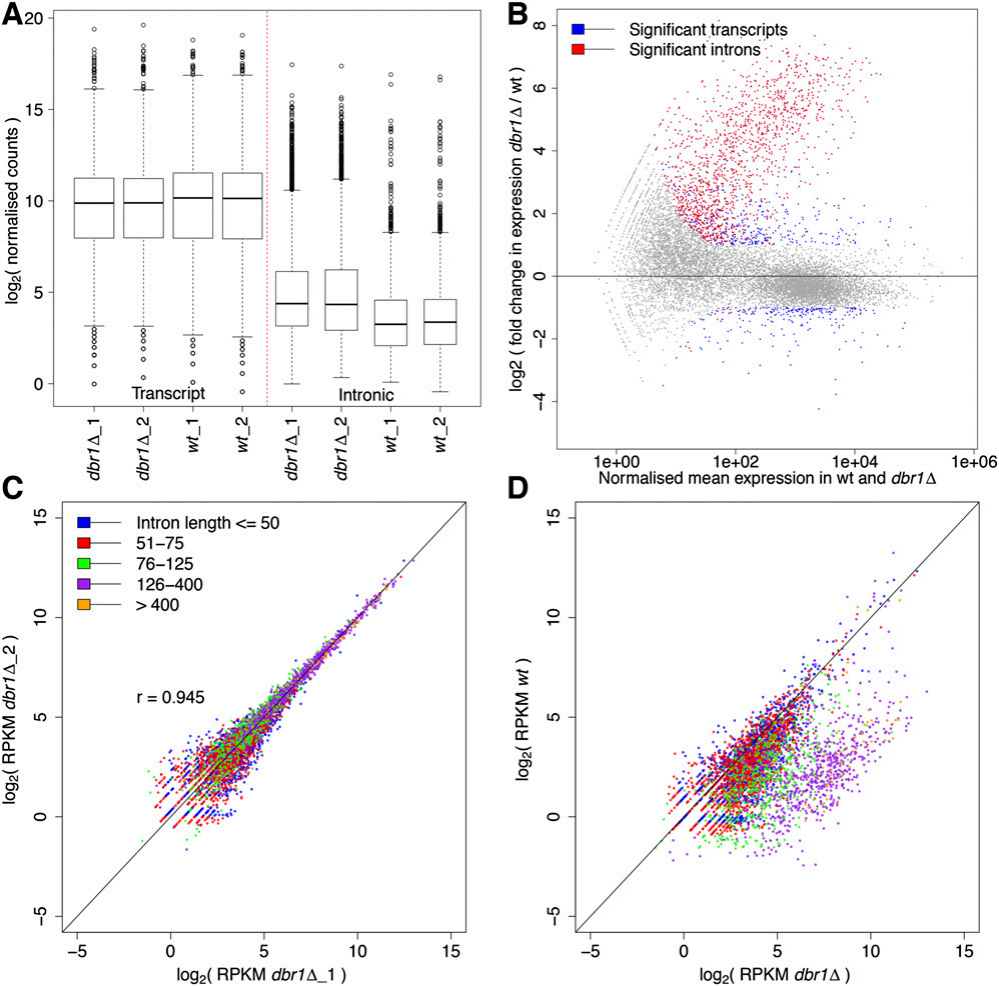
Increased, length-biased intronic expression in *dbr1Δ* cells. (*A*) Box plot showing transcript and intronic expression in *dbr1Δ* and wild-type cells. Intronic expression is significantly higher in *dbr1Δ* (*P* < 2.2 × 10^−16^, Wilcoxon rank sum test). (*B*) MA plot showing differentially expressed introns (red) and transcripts (blue) (DESeq; adjusted *P* < 0.05 and absolute fold change > 2). (*X*-axis) Mean of normalized counts. (*C*) Reproducibility of intronic expression between *dbr1Δ* biological replicates (each dot represents one of 5361 introns; [r] Pearson’s correlation coefficient). Introns were binned according to length as indicated in color legend. (*D*) Comparison of intronic expression between *dbr1Δ* and wild type with introns binned as in *C*. Only one comparison is shown; the other biological replicates produced the same trends. The higher intronic expression in *dbr1Δ* cells shows a strong length bias (*P* < 2.2 × 10^−16^, Wilcoxon rank sum test).

We sequenced the transcriptomes from both *dbr1Δ* and wild-type fission yeast during cell proliferation. The expression data of transcripts, exons, and introns were highly reproducible between biologically repeated experiments (Supplemental Fig. 1). Global transcript levels were similar in wild-type and *dbr1Δ* cells (Fig. 2A). Relative to wild-type transcript levels, 347 and 233 transcripts were significantly increased and decreased, respectively, in *dbr1Δ* cells (Fig. 2B; Supplemental Table 1). Notably, ∼57% of the increased transcripts were noncoding RNAs. The remaining increased transcripts were enriched for Gene Ontology (GO) terms related to mitochondrial translation such as tRNAs (Supplemental Table 2). Even under the strict cutoff applied, 50% of all mitochondrial genes were induced (19/38; *P* < 1.4 × 10^−15^, hypergeometric test), including genes embedded within the group-II, self-spliced *cox1 and cob1* introns (Merlos-Lange et al. 1987). The corresponding lariat intermediates increased in *dbr1Δ* cells and were validated by lariat-specific RT-PCR, showing that these self-spliced introns also depend on Dbr1 in vivo (Supplemental Note 1). The decreased transcripts were enriched for GO categories related to ribosome biogenesis (Supplemental Table 2), including most intron-embedded snoRNAs (6/10; *P* < 2.4 × 10^−7^, hypergeometric test). Intronic snoRNAs are known to depend on Dbr1 for processing in budding yeast (Ooi et al. 1998). These data raise the possibility that protein translation is compromised in the absence of Dbr1. We therefore examined translational profiles in *dbr1Δ* cells. The polysome-to-monosome (P/M) ratio was significantly higher in *dbr1Δ* (1.83) compared to wild type (1.14) (Supplemental Fig. 2). This difference could reflect more efficient translational initiation or compromised translational elongation in *dbr1Δ* cells, either of which would lead to increased ribosomal occupancy along transcripts. Differences in translational initiation are expected to be reflected in different phosphorylation levels of S6 and eIF2α proteins (Lackner and Bähler 2008). However, no such differences were evident between *dbr1Δ* and wild type (Supplemental Fig. 2). Taken together, these findings suggest that translation is compromised in the absence of Dbr1, most likely at the level of elongation.

In contrast to most transcripts, intronic expression in *dbr1Δ* was increased compared to wild type (Fig. 2A). Relative to wild-type intron levels, ∼25% of the introns were significantly increased in *dbr1Δ* under a stringent cutoff (1399/5361) (Supplemental Tables 1, 2), while only 27 introns were decreased (Fig. 2B). While intronic expression was highly reproducible between the biologically repeated *dbr1Δ* experiments (Fig. 2C), many introns showed a pronounced shift toward higher expression in *dbr1Δ* compared to wild type (Fig. 2D). Notably, this increased intronic expression was heavily biased for long introns, with the longest introns showing the greatest extent of increase (Fig. 2D; Supplemental Fig. 3). This bias could reflect floating intron lariats, i.e., the detached lariats that accumulate in the absence of Dbr1 (Fig. 1C), and/or lariat structures still bound to their downstream exons due to inefficient splicing (Hilleren and Parker 2003). In either case, the bias originates mainly from long introns that contribute disproportionately to intronic signals (Fig. 1F). Lariats from long introns are expected to be enriched for technical reasons: 93% of the *S. pombe* introns are shorter than the average insert size used for sequencing (∼200 bases), and ∼36% of the introns are even smaller than the 49-bp read length used. Thus, lariats from short introns were not as efficiently recovered as those from long introns (Fig. 5D, see below).

**Figure 3.**
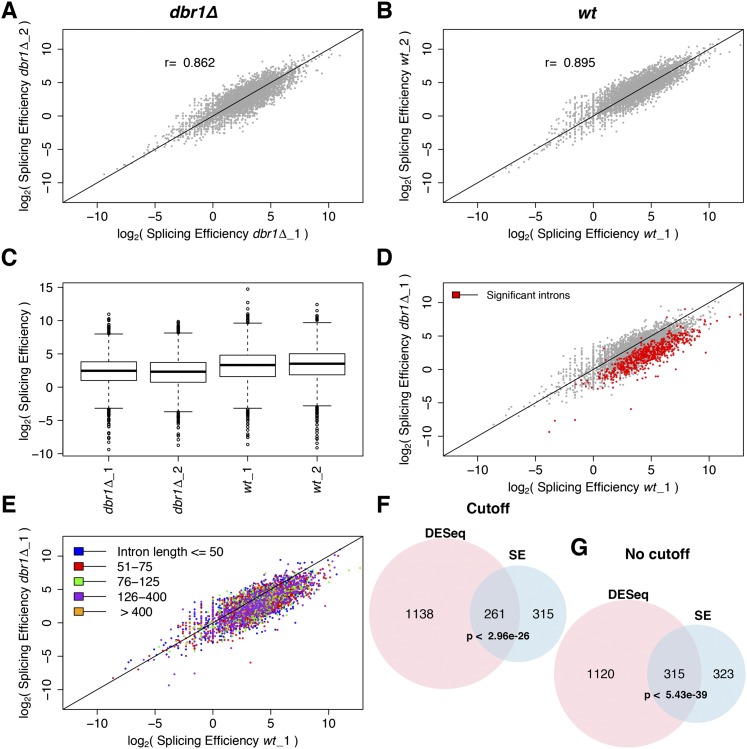
Splicing efficiency is decreased in *dbr1Δ* cells. (*A*,*B*) Correlation of splicing efficiency (SE) between biological replicates in *dbr1Δ* (*A*) and wild type (*B*). Each dot represents one of 5361 introns; (r) Pearson’s correlation coefficient. (*C*) Box plot showing SE in *dbr1Δ* and wild-type cells (*P* < 2.2 × 10^−16^, Wilcoxon rank sum test). (*D*) Comparison of SE between *dbr1Δ* and wild type. (Red) 638 introns showing significant changes in SE (CMH test; *Q* < 0.05). (*E*) As in *D* but with introns binned according to their size as indicated in color legend. (*F*,*G*) Overlap between introns with lower SE and introns with higher expression (DESeq), both with and without fold-change cutoff. The indicated *P*-values for overlaps are based on a hypergeometric test.

To assess differential intronic expression independently of lariats, we analyzed the splicing efficiency (SE). SE is determined using diagnostic exon-intron (Fig. 1A) and exon-exon junction (Fig. 1C) reads that only originate from pre-mRNAs and mRNAs, respectively. SE was highly reproducible between biological replicates for both *dbr1Δ* and wild type (Fig. 3A,B). The overall SE was notably lower in *dbr1Δ* compared to wild type (Fig. 3C). Moreover, there was a pronounced shift for many introns toward lower SE in *dbr1Δ* (Fig. 3D). Unlike intronic expression based on DESeq (Fig. 2D), this lowered SE showed no bias toward long introns (Fig. 3E; Supplemental Fig. 4). As only the DESeq-based analysis includes signals from floating lariats and/or from lariats still bound to their downstream exons, this finding further supports the interpretation above that lariats from long introns led to the bias in intronic expression. The introns showing increased abundance based on DESeq strongly overlapped with those showing decreased SE in *dbr1Δ* cells (Fig. 3F,G; Supplemental Table 2). Thus, there was good agreement between the two methods, despite the DESeq analysis being “contaminated” with lariat reads. Notably, these results together indicate that lariat accumulation and compromised intron turnover in the absence of Dbr1 negatively affects the splicing efficiency of hundreds of introns.

### Generation of lariat databases by LaSSO

**Figure 4.**
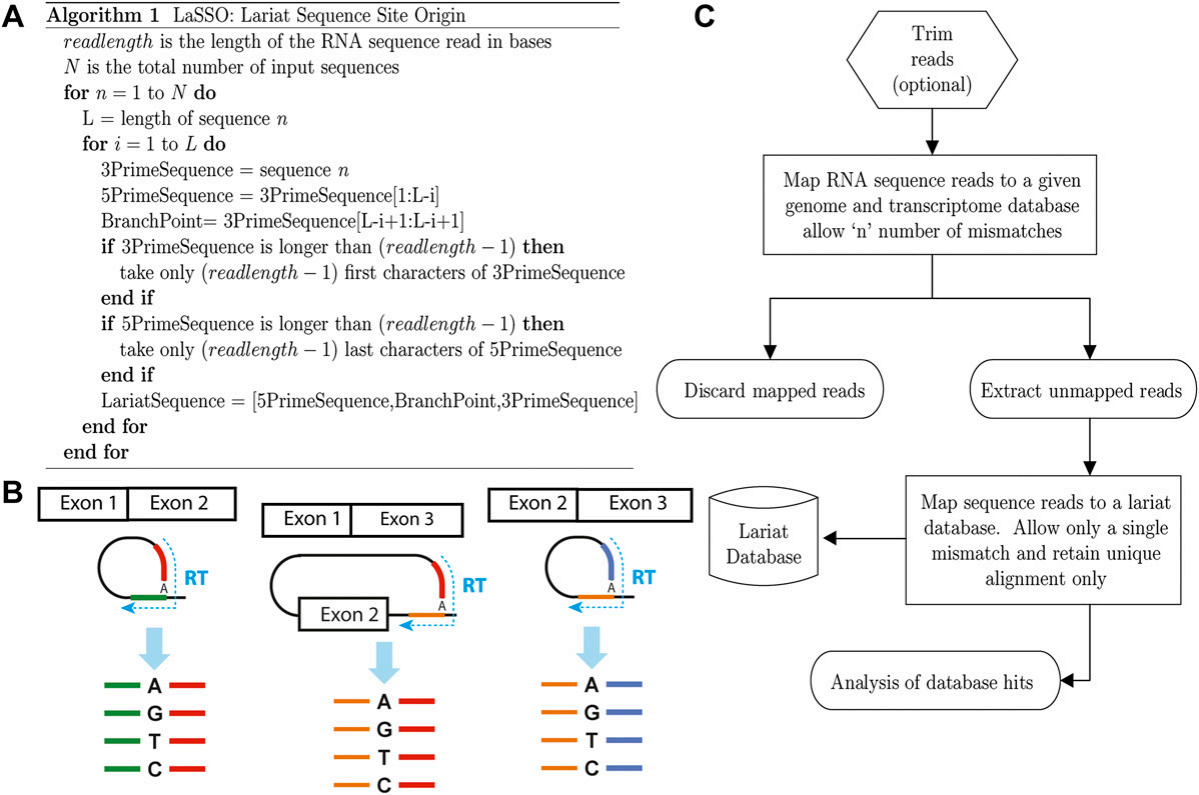
LaSSO (Lariat Sequence Site Origin), an algorithm to build a lariat database along with workflow to identify lariat reads from RNA-seq data. (*A*) The algorithm pseudocode. LaSSO takes a given intron sequence of length “L” and uses the first “read length-1” bases of this intron as the 3′-lariat segment (if shorter, the whole sequence is used). To generate the 5′-lariat segments, accounting for all possible combinations of lariat structures, LaSSO iteratively produces all possible segments by selecting each base at a time as the putative branch point. LaSSO works from the 3′ end of the intronic sequence toward the 5′ end, until it reaches the first intronic base. LaSSO takes only the last read length-1 bases of the 5′-lariat segment (if shorter, the whole sequence is used again). LaSSO then concatenates the 5′ segment, the branch point, and the 3′ segment of the lariat sequence, yielding a diagnostic lariat signature. To generate all possible exon-skipping lariat sequences for a given transcript, the input sequence and algorithm were slightly altered. Briefly, considering a gene with two introns and three exons, only a single skipping event can occur. Therefore, the input sequence is the upstream intron with the downstream intron attached to its 3′ end. To avoid database redundancy, the algorithm iterates L times, where L only refers to the length of the downstream intron, not the combined introns. Thus, the 5′ segment of the skipping lariat sequence is generated from the downstream intron, while the 3′ segment of the skipping lariat always corresponds to the 5′ end of the upstream intron. For more than two introns, all possible skipping events are considered, i.e., S_n_ = (I−1) × I/2 (I: number of introns, S_n_: number of skipping events). (*B*) Scheme for all possible lariat signatures accounted for by LaSSO. Intron excision results in diagnostic cDNA products upon reverse transcription, where the sequence upstream of the branch point precedes the 5′ end of the intron (resulting in 5′- and 3′-lariat segments, respectively). (Green) 5′-lariat segment from upstream intron; (red) 3′-lariat segment from upstream intron; (orange) 5′-lariat segment from downstream intron; (blue) 3′-lariat segment from downstream intron. (*C*) Lariat detection workflow (see main text for details).

We developed the LaSSO algorithm to identify diagnostic lariat reads from RNA-seq data. LaSSO considers each base in a given intronic sequence as a potential branch point and builds all possible lariat structures accordingly, including all theoretically possible exon-skipping events within the corresponding transcript (Fig. 4A,B; Supplemental Fig. 5). The algorithm reflects the ability of the reverse transcriptase to read through the 2′–5′ phosphodiester bond (Ohi et al. 2007; Gao et al. 2008), which results in unique cDNA products that join together two separate intronic segments in reverse order (Fig. 1F,G; Gao et al. 2008; Taggart et al. 2012): the 5′ region upstream of the branch point, which precedes the 5′ end of the intron. Moreover, the reverse transcriptase often mutates the branch-point base from adenine to any other nucleotide (Figs. 1G, 4B; Gao et al. 2008; Taggart et al. 2012; Awan et al. 2013).

Figure 4C shows the analysis pipeline based on LaSSO. RNA-seq reads were first trimmed to remove any adaptor sequences. Given the small size of fission yeast introns relative to sequence-library insert size and read length, it is possible that some short inserts were contaminated by adaptor sequences and hence failed to align. The trimming may therefore enhance the identification of short lariats. The trimmed reads were then aligned to the *S. pombe* genome and transcriptome using Bowtie (Langmead et al. 2009), allowing three mismatches. The unmapped reads were extracted (Supplemental Table 3) and aligned to the lariat database; to account for the likely mutation at the branch point by the reverse transcriptase, LaSSO allows one mismatch (Figs. 1G, 4B). Reads aligning to the lariat database were then used for genome-wide mapping of branch points and lariat analyses.

### Characterization of lariats and branch-site sequence

**Figure 5.**
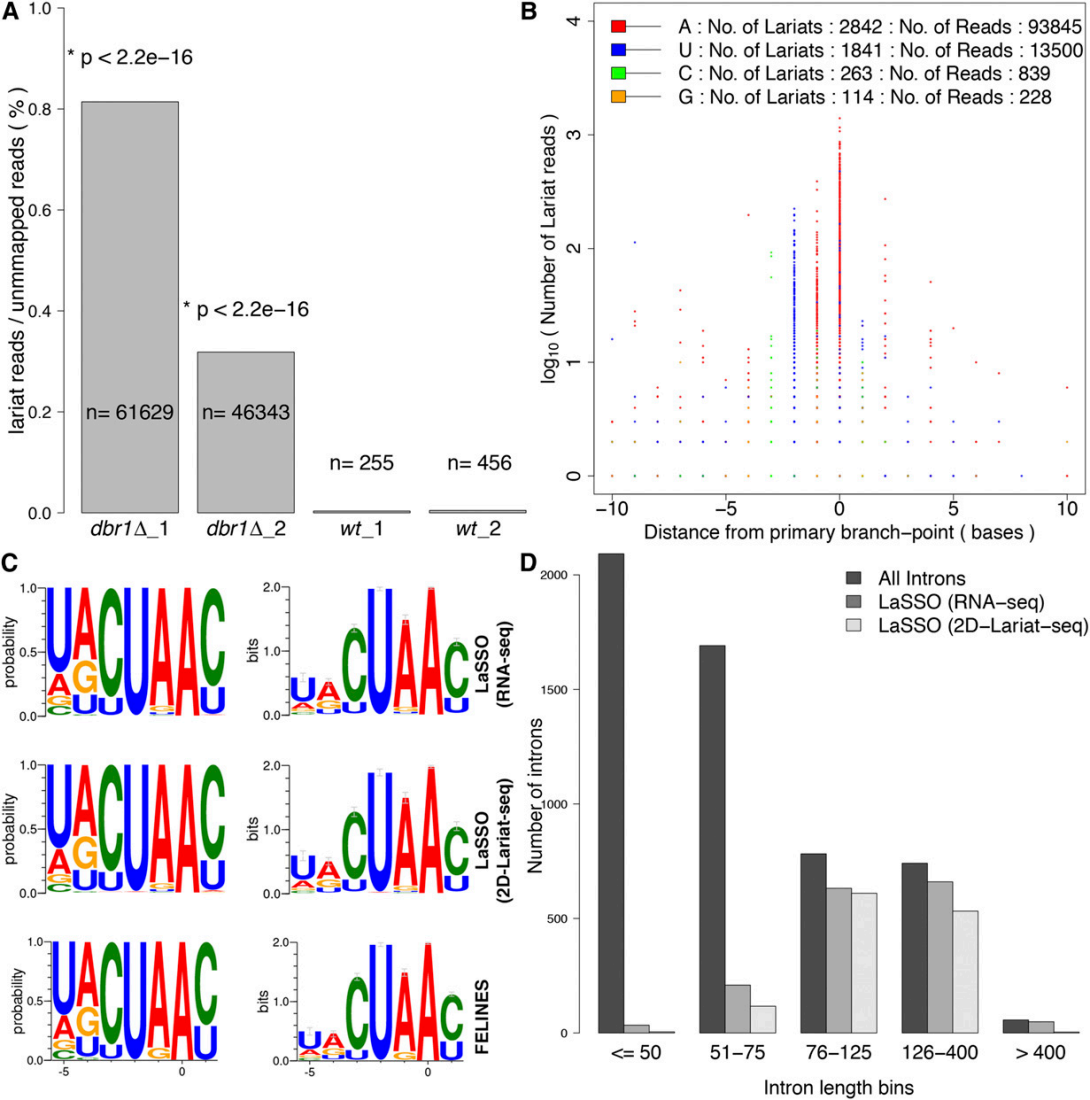
Characterization of lariat branch points and branch-site sequence. (*A*) Proportion of lariat reads relative to total number of reads not mapped to genome or transcriptome. Absolute numbers in each sample are indicated, along with *P*-values (Fisher’s exact test). (*B*) The base (color-coded as indicated) and position (*x*-axis) of each branch point identified as a function of read number supporting it (*y*-axis). The primary branch point is placed at position zero on the *x*-axis. The numbers of lariats and supporting reads are indicated on *top*. Only branch points located within 10 bases up- (negative values) or downstream (positive values) from a primary branch point are shown. (*C*) Consensus branch-site sequences around the primary branch point as probability (*left*) and bits (*right*), plotted using WebLogo (Crooks et al. 2004) (default settings except for compositional adjustment, with GC content set to 30%). (*Top* panels) Using LaSSO based on 1236 introns from our data; (*middle* panels) using LaSSO based on 930 introns from 2D-Lariat-seq data (Awan et al. 2013) that were supported by ≥3 lariat reads; (*bottom* panels) using FELINES for the same set of 1236 introns detected in this study. (*D*) Number of introns of different sizes for which lariat reads were detected by LaSSO when no read-number threshold was applied: 1584 lariats for our data, 1268 lariats for 2D-Lariat-seq data by Awan et al. (2013). Introns were binned according to their size as indicated (5361 introns in total).

We identified 108,683 diagnostic lariat reads that aligned to the *S. pombe* lariat database. As expected, *dbr1Δ* cells were highly enriched for these lariat reads compared to wild type (Fig. 5A). Despite this enrichment, lariat reads corresponded only to a small fraction of the mappable reads (Supplemental Table 3). Most lariat reads (∼99.8%) originated from within 1655 single introns, defining 5060 distinct lariats. These data indicate the presence of multiple branch points per intron. We ranked the branch points for each intron by the number of mapped lariat reads and defined the one supported by the highest read number as the primary branch point. We then aligned all other branch points relative to the primary branch point, highlighting the corresponding base (Fig. 5B). This analysis provided the following results: (1) Adenine was the predominant primary branch-point base; (2) neighboring branch points were clustered within a few bases of the primary branch point, suggesting a certain “fuzziness”; and (3) together, the clustered branch points formed a sequence motif similar to the branch-site consensus, YURAY (Mertins and Gallwitz 1987; Drabenstot et al. 2003). The fuzziness around the primary branch point could reflect sequencing or informatics noise, imprecision in branch-point selection by the spliceosome, or inaccuracies introduced by the reverse transcriptase during transcription of the 2′–5′ phosphodiester bond. The latter explanation seems most likely, given the known high mutation rates and base skipping during reverse transcription of the branch point (Supplemental Note 1; Supplemental Fig. 6; Gao et al. 2008; Taggart et al. 2012; Awan et al. 2013). The finding that the clustered branch points make up branch-site consensus sequences further supports the interpretation of base skipping by the reverse transcriptase. We therefore suggest that the clustered branch points primarily reflect reverse-transcription errors around the main adenine branch points. However, we cannot rule out some cellular imprecision during the transesterification reaction.

Given that our data suggest that few, if any, bases other than adenine serve as branch points, we ignored all non-adenine branch-point reads for the further analyses below. Considering only the 93,845 adenine branch-point reads, we identified 2842 distinct lariats originating from 1584 introns. We adjusted the LaSSO algorithm to consider only adenine as possible intronic branch points (Supplemental Fig. 7). Even when considering only adenine branch points, limited fuzziness by neighboring branch points remained within ∼2 bases. We therefore developed a clustering approach to group neighboring branch points as follows. The primary branch point was defined by the highest read number; if read numbers between neighboring branch points differed by <10, however, the one closer to the intronic 3′ end was selected as the primary branch point (when neighboring branch points differed by >10 reads, the one closer to the 3′ end typically showed the higher read number). If branch points were >1 base apart, multiple branch points were reported. To err on the conservative side, we only used the branch points supported by ≥3 lariat reads, which originated from 1236 nuclear introns and one mitochondrial, self-spliced intron (*cob1*). Based on these data, we generated a consensus branch-site sequence for nuclear-encoded genes (Fig. 5C; Supplemental Table 5).

We selected 18 lariats of different lengths and numbers of diagnostic lariat reads for independent validation. We confirmed 17 of these 18 lariats by lariat-specific RT-PCR, followed by Sanger sequencing (Supplemental Fig. 8; Supplemental Table 6). One of the validated lariats was from the self-spliced *cox1* intron, for which uracil appears to be the primary branch point (Supplemental Note 1).

The numbers of lariat and exon-exon junction reads showed only a poor correlation (Supplemental Fig. 9). This finding suggests that the number of lariat reads does not quantitatively reflect the number of splicing events. As indicated by the expression data, short lariats were not recovered as efficiently as long ones. Although we trimmed the reads prior to alignment to enhance identification of short inserts, we could not identify branch points for most introns shorter than ∼75 bases, even when no three-read threshold was applied (Fig. 5D).

The median distance between the branch point to the 3′ end of the intron was 12 bases. More than one branch point was only evident for 89 introns using the criteria above. Compared to primary branch points, alternate branch points were covered with a significantly lower number of reads and were also located closer to the 5′ end of the intron (*P* < 2.2 × 10^−16^ and *P* < 5.5 × 10^−9^, respectively; Wilcoxon rank sum test). Intron lengths and numbers of branch points did not correlate. We conclude that a single, primary branch point is used for most splicing reactions under the standard condition analyzed.

### LaSSO outperforms other algorithms

The consensus branch-site sequence derived by LaSSO was nearly identical to the one predicted by FELINES (Fig. 5C; Drabenstot et al. 2003), corresponding to the reported “YURAY” motif (Supplemental Note 2). Overall, there was ∼91% agreement between LaSSO and FELINES across data sets (Supplemental Note 2), suggesting that computational prediction of branch sites in yeast is quite robust. As LaSSO analyzes empirical data, however, it could uncover branch sites more accurately (Supplemental Note 2).

A recent study utilized a 2D-gel lariat purification approach (2D-Lariat-seq) in *S. pombe dbr1Δ* cells to map branch points (Awan et al. 2013). Using a read-split algorithm based on a previous study (Taggart et al. 2012), they recovered 37,008 lariat reads that were mapped to 817 annotated and novel introns (Awan et al. 2013). When applying LaSSO to their data set, we recovered 97,072 lariat reads, providing a >2.5-fold increase in sensitivity. This improved detection was achieved despite only interrogating annotated introns, not the whole genome as in the original study (Awan et al. 2013). Applying our clustering approach, we mapped branch points for 1268 introns, of which only 813 were identified in the original study. On the other hand, we missed only four branch points detected originally (Awan et al. 2013), because the corresponding five reads had >1 mismatch and did not meet our mapping criteria (Supplemental Table 7). The consensus branch-site sequence was highly similar again when applying LaSSO on the 2D-Lariat-seq data (Fig. 5C). Note that the underrepresentation of short lariats was also apparent, and even more pronounced, in the 2D-Lariat-seq data set (Fig. 5D). We conclude that LaSSO is more accurate than the theoretical prediction algorithm and can recover more lariats than an alternative approach using sequence data (Awan et al. 2013).

### Exon-skipping events

We also looked for lariats diagnostic for exon-skipping events. In fission yeast, there is little evidence for alternative splicing via intron retention or exon skipping (Habara et al. 1998; Moldon et al. 2008; Wilhelm et al. 2008; Rhind et al. 2011; Awan et al. 2013). Only 271 of 108,683 lariat reads represented putative skipping events (0.25%). Of these, only 82 reads marked adenine as the branch point, representing 48 distinct exon-skipping events, including seven instances where two exons were skipped in a single event. Only 18 of these 48 skipping events were independently supported by a small number (median = 1) of exon-exon junction reads that bridged non-neighboring exons. The independent evidence based on lariat and junction reads indicates that these skipping events represent real cellular splicing events. We independently confirmed five out of five representative exon-skipping lariats by lariat-specific RT-PCR, and four out of these five were also confirmed by Sanger sequencing (Supplemental Fig. 8; Supplemental Table 6). For ∼69% of cases (33/48) (Supplemental Table 8), the branch points identified for the skipping events were identical to the one identified for the canonical splicing event, which links neighboring exons. We conclude that skipping events usually utilize the primary branch point of the downstream intron.

Further analysis of all exon-skipping events revealed that the number of supporting lariat reads was generally low (median = 1), with only six events exceeding our three-read threshold. In all cases, the canonical splicing events were supported by much higher read numbers compared to the corresponding skipping event (median = 30.5; *P* < 6.9 × 10^−14^) (Supplemental Table 8). Only 14 of 48 skipped sequences were divisible by three, as expected by chance, and the remaining events are predicted to change the reading frame (Magen and Ast 2005). The branch-site sequence of the upstream intron determines the likelihood for exon-skipping events (Haraguchi et al. 2007). We therefore examined the branch-site sequences for all upstream and downstream introns implicated in exon skipping, but no sequence differences compared to the consensus were apparent (Supplemental Fig. 10). Together, these data suggest that most exon-skipping events have no biological role under the standard condition analyzed but might reflect splicing errors.

The only evidence for exon skipping in fission yeast has been documented recently in the 2D-Lariat-seq study (Awan et al. 2013). They reported 23 cases of exon-skipping events, only eight of which were supported by 12 lariat reads in total; although five of these events were validated by RT-PCR, the remaining events were only detected by a peak-calling algorithm. Applying LaSSO on the 2D-Lariat-seq data, we recovered 174 reads that marked 94 potential skipping events. In the 2D-Lariat-seq data, the skipping events displayed similar characteristics as in our data: Only 14 skipping events were supported by ≥3 lariat reads, 20 were supported by junction reads in our data, and only 32 of 94 skipped sequences were divisible by three. Just one of the four events reported to be conserved in human (Awan et al. 2013) was identified by LaSSO (*alp41*) (Supplemental Table 10). Given the low diagnostic read numbers, further experiments will be required to test whether these exon-skipping events have cellular functions or represent splicing errors destined to degradation (Magen and Ast 2005). In budding yeast, some exon-skipping RNAs are targeted by quality-control pathways (Egecioglu et al. 2012). Regardless of their potential to form functional transcripts in fission yeast, our data demonstrate that LaSSO is effective for the identification of exon-skipping lariats.

### Lariats and exon skipping in human

We also applied LaSSO to the more complex human genome, where introns are typically much longer and branch sites are degenerate (Kol et al. 2005). To this end, we generated a comprehensive human lariat database, based on adenine as the branch point, against which we aligned more than 592 million unmapped reads derived from 16 human tissues; this data set is known to contain lariat reads (Taggart et al. 2012). To err on the side of caution, we applied stringent filtering criteria (see Methods), resulting in the identification of 586 adenine-based lariat reads. These reads mapped to 287 annotated introns (Supplemental Table 11; Taggart et al. 2012) and 34 exon-skipping events (Supplemental Table 12). Owing to the differences in methods, there was only little overlap between the branch points identified here and by Taggart et al. (2012) (Supplemental Note 3). LaSSO detected more accurately the location of branch points (Supplemental Fig. 11; Supplemental Note 3), and it identified branch points implicated in exon skipping (Supplemental Fig. 12C).

Plotting the positions of all branch points as a function of the corresponding intron length revealed two distinct types: proximal branch points close to the 3′ end of the intron, whose intronic positions showed no dependency on intron length, and more distal branch points, whose intronic positions showed dependency on intron length (Supplemental Fig. 12). Similar distal branch points were evident in fission yeast which, unlike in the human data, were supported by high read numbers and consensus branch-site sequences (Supplemental Fig. 12). In the human data set, but not in fission yeast, most exon-skipping events seemed to utilize the more distal type of branch point, although the numbers of diagnostic reads was very low (Supplemental Fig. 12C,D).

Since the human data set has been generated in the presence of debranching activity, the ≥3 lariat read cutoff inevitably resulted in discarding most of the putative branch points. However, regardless of whether a read cutoff was applied or not, the human branch site was highly degenerate (Supplemental Table 9), in accordance with previous reports (Kol et al. 2005). A consensus branch-site sequence was evident only with a subset of proximal branch sites (Supplemental Fig. 13; Supplemental Table 9), reminiscent of the previously reported consensus sequence (Gao et al. 2008). We conclude that LaSSO is also effective for identifying branch points in more complex genomes, although reducing debranching activity is important for a high signal-to-noise ratio.

## Discussion

We developed a genome-wide approach to capture transient RNA lariat intermediates that are rapidly degraded by the cell under normal conditions. This approach enables an experimental definition of intronic branch points and intron validation, furthering our view of splicing and intron processing.

RNA-seq of *dbr1Δ* cells revealed that lariat accumulation affects gene regulation, most notably global translation. These results are consistent with the reported high toxicity of lariat accumulation (Nam et al. 1997) and a role for Dbr1 in processing of snoRNAs (Ooi et al. 1998) and mirtrons (Flynt et al. 2010). These findings may reflect the tight coordination and integration of gene regulation at multiple levels, which leads to systemic changes when intron degradation is perturbed. Furthermore, we show that lariat accumulation also severely compromised splicing efficiency of numerous introns. This intriguing finding raises the possibility of a feedback from floating lariats to the splicing machinery to prevent further lariat accumulation. A reduction in splicing efficiency was also observed in the *ACT1* locus of debranching-deficient budding yeast cells, and the accumulated *ACT1* intron may slow down later stages of the splicing reaction (Salem et al. 2003). Decreased recycling of spliceosomal RNAs and proteins, which might not be released from stable lariats, could lead to the decreased splicing efficiency (Arenas and Abelson 1997; Martin et al. 2002; Hilleren and Parker 2003). A Dbr1-dependent RNA degradation pathway prevents the accumulation of splice-defective lariat intermediates that are otherwise exported to the cytosol for degradation, thus functioning as a quality-control mechanism for splicing (Hilleren and Parker 2003). Most fission yeast proteins involved in splicing do themselves contain introns (100 of the 154 proteins with GO terms related to splicing), which could also contribute to the global decline in splicing efficiency via negative feedback.

Another intriguing finding was the up-regulation of numerous noncoding RNAs in *dbr1Δ* cells. Many of these RNAs do contain introns (data not shown), which therefore may be targeted by Dbr1. We also observed that the absence of Dbr1 affected mitochondrial gene expression and splicing. Both RNA-seq and lariat-specific RT-PCR provided evidence for the accumulation of the corresponding lariat intermediates in *dbr1Δ* cells, strongly suggesting that group II, self-spliced mitochondrial introns depend on Dbr1 in vivo. Thus, it is likely that besides nuclei and cytosol (Hilleren and Parker 2003), Dbr1 also functions in mitochondria.

LaSSO provides a powerful, unbiased approach to identify lariat intermediates and to precisely map branch points on a genomic scale. More branch points were identified by LaSSO compared to 2D-Lariat-seq (Awan et al. 2013), both with read cutoff (1236 and 930, respectively) and without (1584 and 1268, respectively); LaSSO therefore circumvents the need for laborious 2D-PAGE isolation and purification of lariats prior to sequencing. LaSSO outperformed the FELINES algorithm for theoretical branch-site prediction (Drabenstot et al. 2003), and it provided higher sensitivity and improved accuracy than bioinformatics approaches applied in previous sequencing studies (Taggart et al. 2012; Awan et al. 2013). LaSSO effectively uncovered exon-skipping lariats both in fission yeast and in human. In fission yeast, some of the skipping events were supported by independent exon-exon junction reads and could represent true alternative transcript isoforms. Nevertheless, given the exceedingly low frequency of most skipping events in two independent studies, involving just a few instances among the millions of cells sequenced, it seems likely that the majority of these skipping events are simply a consequence of aberrant splicing, which increase under heat-stress conditions (Awan et al. 2013). Consistent with this interpretation, skipping events are greatly increased in mutants defective for RNA quality-control pathways (DA Bitton and J Bähler, unpubl.). Further work will be required to establish whether exon skipping has any biological function in fission yeast, perhaps limited to specialized conditions.

Several technical issues affected the performance of LaSSO. Given the short fission yeast introns, the size selection during sequence library preparation, combined with the short RNA-seq reads, resulted in a bias against recovery of short lariats. It is possible that adjustments to the sequencing protocol, such as the one used for small RNA profiling, could improve detection of small lariats. This bias will be less relevant for organisms like human, which generally have much larger introns, although the shortest human introns may still be affected (“minimal” introns) (Yu et al. 2002). Another issue was the aggregation of branch points next to the primary branch point that was most likely introduced by reverse-transcriptase errors. Our rigorous clustering approach helped to deal with this issue. The reduced efficiency of reverse transcription through the 2′–5′ bond often resulted in an underestimation of the lariats. Thus, lariat reads should be used only as diagnostic reads rather than for quantification of splicing events or the extent of lariat accumulation.

LaSSO is a versatile algorithm that allows RNA-seq input from any organism, thus allowing multiple applications. Effectively, it allows detection of any 2′–5′ phosphodiester link in a given sequence, but the algorithm does depend on the input database used. For example, to identify additional cryptic or alternative splice sites, an intronic sequence could be analyzed with its corresponding upstream or downstream exon sequence. Alternatively, to seek for naturally occurring 2′–5′ phosphodiester bonds in mature RNAs, complete transcriptome sequence could be used as an input. In addition, LaSSO could readily be applied to detect novel splicing events by partitioning the genome with a sliding window while ignoring known annotations.

An advantage of LaSSO is that it can consider all possible bases in a given intron. This circumvents making assumptions on branch-site positions and sequences. However, it may result in exceedingly large lariat databases when numerous, large introns, such as those of human, are considered. We provide RNA-seq and RT-PCR evidence for uridine as the branch-point base within the *cox1* intron of the mitochondrial genome. However, the data provided here indicate that adenine is the predominant branch-point base in fission yeast, and exceptions might reflect technical artifacts during reverse transcription.

We generated a human lariat database that only considers adenine as branch points within introns, but also accounts for all possible branch points generated by exon skipping. Following alignment of RNA-seq data against this human lariat database, we applied stringent filtering criteria, which inevitably also discarded some real lariat signals. For a higher signal-to-noise ratio, working with debranching-deficient cells will be important. An siRNA knockdown in human cells effectively reduces 80% of *DBR1* transcript levels without affecting cell viability (Ye et al. 2005), which could readily be exploited in future studies. Despite the presence of debranching activity, LaSSO identified numerous branch sites, including those diagnostic for exon-skipping events. Our analysis is consistent with the human branch-site consensus being highly degenerate; previous consensus sequences were assembled using only a subset of proximal branch sites (Gao et al. 2008), as was recapitulated here. LaSSO suggested putative distal branch points whose intronic positions depended on intron length in both fission yeast and human. Intriguingly, in the human data set, most exon-skipping events seemed to utilize this distinct type of branch point, but the numbers of diagnostic reads were very low. It is not clear whether the distal branch points reflect an experimental artifact or a novel biological aspect, e.g., an alternate, closer branch point to facilitate lariat formation for long introns. It should be interesting to explore whether these distal branch points have any biological relevance in the cell.

## Methods

### Strains and yeast techniques

The *dbr1Δ* strain was obtained from the Bioneer deletion collection v.2.0 (Kim et al. 2010). The deletion mutant strain was PCR-verified and exhibited slow growth in YES media. When back-crossed to wild type, the slow-growing phenotype cosegregated with the deletion marker. Strain ED668 (*h+ ade6-M216 ura4-D18 leu1–32*) contains the same genetic background as the *dbr1Δ* strain (except for *dbr1Δ* deletion) and was used as a wild-type control in these studies. Translational profiles (Lackner et al. 2012) and Western blots (Rallis et al. 2013) were performed as previously described.

### RNA isolation and sequencing

Biological replicates were grown and processed separately for all the following steps. Two biological replicates of wild-type (ED668) and *dbr1Δ* cultures were grown in YES (yeast extracts plus supplements) media at 32°C until they reached a concentration of 5,600,000 cells/mL. Cells were harvested and total RNA was isolated by hot-phenol extraction, and RNA quality was assessed on a Bioanalyzer instrument (Agilent). Total RNA was treated with DNase (Turbo DNA-free by Ambion), and thereafter, 4 μg was treated with a beta version of Ribo-Zero Magnetic Gold Kit (Yeast) to deplete rRNAs. RNA-seq libraries were prepared from rRNA-free RNA using a strand-specific library preparation protocol based on an early version of the Illumina TruSeq Small RNA Sample Prep Kit. In brief, rRNA-depleted RNA was fragmented to an average size of ∼200 nt. Fragmented RNA was 3′-de-phosphorylated with Antarctic phosphatase and 5′-phosphorylated with polynucleotide kinase; this treatment prepares RNA fragments for subsequent ligation of Illumina RNA adaptors to their 5′ and 3′ ends using a 3′-RNA ligase and a T4 RNA ligase, respectively. First-strand cDNA was produced using a primer specific for the Illumina 3′-adaptor. The library was amplified with 15 PCR cycles using primers specific for the Illumina adaptors and purified using SPRI-beads (Agencourt, Beckman Coulter). Library size distributions and concentrations were determined on a Bioanalyzer (Agilent). RNA-seq libraries were sequenced on an Illumina HiSeq 2000 instrument at the Core Facility of the Huntsman Cancer Institute (University of Utah).

### Genome level alignments and annotation

Sequence reads of 49-base length originating from each sample were aligned, using Bowtie 0.12.7 (Langmead et al. 2009), to the *S. pombe* genome sequence (Ensembl *S. pombe*, Build EF1, version 13) (Flicek et al. 2014) and to the corresponding exon-exon junctions database. Up to three base-pair mismatches were allowed. Reads that matched multiple loci were removed from further analysis, and the resultant alignment files were processed to generate “pile-ups” against each chromosome (Supplemental Table 2). Unmapped reads were used for lariat mapping as described in the main text.

### Exon-exon junctions

Searches were performed against the genome sequence combined with a data set of known exon-exon junctions as defined by Ensembl *S. pombe,* release 13. To ensure that a 49-base read mapped to a splice junction, only the last 43 bases of the first exon and the first 43 bases of the second exon were considered (if the exon exceeded length 43). In this way, reads that overlapped a junction by less than 6 nt were excluded. Reads that matched to more than one junction or elsewhere in the genome were also discarded.

### Known annotated transcript set

The known annotated set of *S. pombe* transcripts (7022; Ensembl version 13, as before) and all known introns (5361) were interrogated across the four samples (a total of 12,383 loci) (Supplemental Table 2).

### Normalization, fold changes, and differential expression

Differential expression between samples was determined using the DESeq Bioconductor package (Anders and Huber 2010). A cutoff of ±2 fold change and corrected *P*-value < 0.05 were applied to derive a list of differentially expressed genes and introns.

### RNA-seq expression level

Normalized expression levels (E) for individual exons and introns were calculated using Equation 1 as described (RPKM measure) (Mortazavi et al. 2008). Briefly, the number of reads (*R*) detected across a given region at a given sample (*i*) was multiplied by a constant (*C* = 1 × 10^9^) and divided by the total number of reads at that sample (*T*_*i*_) multiplied by the region’s length (*L*).
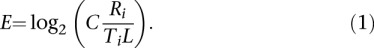
A small constant was added (10^−5^) to all expression values to avoid taking logs of zero. Gene level expression values were summarized using exon data. Sample specific expression levels for all regions interrogated in this study are provided in Supplemental Table 2.

### Splicing efficiency and differential splicing significance

Splicing efficiency (SE) reflects the proportion of spliced mRNA signal relative to pre-mRNA signal. SE is computed by dividing exon-exon junction reads (*JR*) by reads that straddle the exon-intron boundary (only the upstream 5′ exon relative to the intron was considered; EI; Equation 2).
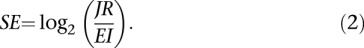
A Cochran-Mantel-Haenszel (CMH) χ^2^ test for repeated test of independence (i.e., biological replicates) was applied to identify statistically significant introns (i.e., introns that display differences in SE between samples). The false discovery rate (*Q*-value) was computed using the Bioconductor *Q*-value package (Storey et al. 2004) and a cutoff of *Q* < 0.05 was applied (Supplemental Table 2).

### Comparisons between studies

The raw sequence data files of the “short” and “long” data sets described in Awan et al. (2013) were downloaded from the Gene Expression Omnibus (GEO) database (accession no. GSE48594). Sequence Read Archive files (SRA) were converted to FASTQ files using the SRA toolkit. To perfectly match the read length described in their study, the reads were preprocessed to trim the first three bases only (no further trimming of adaptor sequences was applied). Thereafter, 40-base-long reads were analyzed using our pipeline as described.

For FELINES comparison, a FASTA file containing 5248 annotated introns with canonical splice donor and acceptor sites (GU and AG, respectively) and of length >20 bp was analyzed using FELINES with default settings.

### Application of LaSSO to a human data set

Using the LaSSO algorithm (Supplemental Fig. 7), we generated a human lariat database that included all possible adenine-based lariat signatures generated from a set of 265,870 nuclear introns in human (Ensembl *Homo sapiens*, version 70). To generate this set, we ordered all annotated exons for each gene in 5′–3′ orientation and considered the gap between two consecutive exons as a putative intron. Note that human mitochondrial genes lack annotated introns. The database also accounted for all possible lariat signatures that could be generated from all possible exon-skipping events in a given transcript, bringing its total size to ∼947.26 GB that accommodate 4,206,823,861 lariat sequence entries. To allow comparison to the study conducted by Taggart et al. (2012), we downloaded the same RNA-seq data set: Illumina Human Body Map 2.0 total RNA from GEO (accession number GSE30611). SRA files were converted to FASTQ files using the SRA toolkit. Reads were preprocessed to trim the adaptor sequences provided in GEO (GSE30611). Using the pipeline described in Figure 4C, we extracted 592,367,167 unmapped reads. Thereafter, the reads were aligned using Bowtie 0.12.7 to the human lariat database that was split into multiple smaller databases to allow parallelization. By tolerating up to a single mismatch and allowing a single alignment, we initially identified 51,469 diagnostic lariat reads. To further reduce the probability of chance matching, we applied a series of stringent filters to remove ambiguous reads: those that were mapped to multiple locations (due to the partitioning of the database), those that were mapped in antisense orientation, those whose mismatched base was not located exactly at the position of the branch point, and those that did not overlap at least 10 bases across the lariat junction. Comparison to lariats identified by Taggart et al. (2012) was performed using the available lariat coordinates (Taggart et al. 2012).

### GO term enrichment

Significant gene lists (increased/decreased) were processed using the “GO Term Finder” algorithm implemented in Perl (Boyle et al. 2004) (cutoff of *P*-value < 0.001, with Bonferroni correction) (Supplemental Table 2).

### LaSSO implementation and databases

The LaSSO algorithm was implemented in R (R Development Core Team 2011) and is freely available at GitHub (https://github.com/dbitton/LaSSO) and in Supplemental File 1. The fission yeast lariat database was constructed based on Ensembl *S. pombe*, Build EF1, version 13, while the human lariat database was generated using Ensembl *Homo sapiens,* version 70.

## Data access

The RNA-seq data sets from this study have been submitted to the NCBI Gene Expression Omnibus (GEO; http://www.ncbi.nlm.nih.gov/geo/) under accession number GSE50246.
